# Climate change communication, adaptation, and mitigation through the arts: a rapid scoping review

**DOI:** 10.3389/fpubh.2026.1804107

**Published:** 2026-10-07

**Authors:** Zoe Moula, Marinela Natsi, Elisabeth Bahr, Sammi Munson, Ian Garrett, Tarah Wright, Ameer Shaheed, Nisha Sajnani

**Affiliations:** 1 Research Division of Care in Long-Term Conditions, Faculty of Nursing, Midwifery & Palliative Care, King’s College London, London, United Kingdom; 2 School of Neuroscience, Institute of Psychiatry, Psychology & Neuroscience, Faculty of Life Sciences & Medicine, King’s College London, London, United Kingdom; 3 Jameel Arts & Health Lab, NYU Steinhardt, New York, NY, United States; 4 Environmental Studies & Sciences, Gonzaga University, Spokane, WA, United States; 5 Department of Theatre, Dance and Performance, York University, Toronto, ON, Canada; 6 Earth and Environmental Science, Dalhousie University, Halifax, NS, Canada; 7 WHO Regional Office for Europe, Copenhagen, Denmark

**Keywords:** climate change communication adaptation mitigation, creative health, environmental arts and humanities, human and planetary health, planetary health challenges: interventions for effective knowledge mobilization for policy-and decision-makers and science communication stakeholder analysis

## Abstract

**Introduction:**

Climate crisis is inherently intersectional; it is a health crisis and a cultural crisis too. Creative and innovative approaches are essential to address the complex and unequal health and wellbeing impacts of human induced climate change. This rapid scoping review aimed to review, synthesise, and analyse empirical studies that implemented arts-based interventions to promote climate change communication, adaptation, and mitigation.

**Methods:**

We used the methodological framework for scoping reviews set out by Arksey and O’Malley and systematically searched five major electronic databases using the PRISMA reporting guidelines.

**Results:**

Sixteen studies met the eligibility criteria and were included in this review. Programmes varied and included environmental art festivals, climate summits, ecological conferences, theater and open-air performances, exhibitions, musical events, fieldtrips, nature conservation and sustainability interventions. Most studies focused on climate change communication, using the arts to raise environmental awareness and translate public health messages more clearly and accessibly. Only four studies focused on climate change adaptation (e.g., arts for coping with climate emotions) and mitigation (e.g., arts for motivating cultural shifts towards low-carbon lifestyles and practices).

**Discussion:**

Arts-based programmes focusing on climate change adaptation and mitigation are disproportionately low compared to climate change communication, signalling missed opportunities to leverage the benefits of the arts. Arts-based programmes delivered to public and outdoor spaces attracted audiences who were not previously interested in the arts and/or climate change, demonstrating the capacity of the arts to reach diverse audiences. Analyses of aesthetic qualities in climate-related artwork suggested that awe-inspiring nature depictions that gently motivate reflexive thinking on environmental issues were viewed more positively compared to artwork that instils didacticism and propagandization. The most impactful arts-based programmes combined emotional and cognitive activation—equipping the public with both the emotional investment and the knowledge needed to take collective environmental action, such as engaging with policymakers. Our findings suggest that the arts can help the public move beyond viewing climate change as an abstract concept, towards envisioning the tangible possibility of a different future. We argue that stronger policy integration, sustained research investment, and enhanced cross-sector collaboration are essential to realising the full potential of the arts in advancing climate communication, adaptation and mitigation, and we propose strategies to advance these priorities.

## 1 Introduction

Climate change is no longer a distant environmental threat; it is an accelerating public health crisis with profound implications for physical health, mental wellbeing, social cohesion, and health equity (1). Rising temperatures, extreme weather events, biodiversity loss, food and water insecurity, and forced displacement are already shaping patterns of disease, psychological distress, and community vulnerability worldwide. These impacts are unevenly distributed, disproportionately affecting children, older adults, Indigenous peoples, low-income areas, and populations living in climate-exposed regions (2–4). As such, climate change is not only a biomedical or environmental challenge, but also a cultural, social, and ethical one.

Despite the growing scientific consensus on the causes and consequences of climate change—including anthropogenic causes connected to industrialization and capitalism—the translation of knowledge into sustained public engagement, adaptive behavior, and collective action remains a persistent challenge. Climate communication efforts often struggle to overcome politicisation, misinformation, emotional disengagement, and the psychological distance people experience when climate change is framed as abstract, future-oriented and/or overwhelming (5). Conventional modes of communication, such as technical reports, can unintentionally widen this distance and limit their reach and effectiveness, particularly among people who do not see climate change as an immediate or personally relevant concern (6).

In response to these limitations, there has been increasing interest in the potential of the arts to play a transformative role in climate change communication, adaptation, and mitigation (7). Across disciplines, the arts have been recognised for their capacity to translate complex information into accessible forms, evoke emotional resonance, foster reflection, and create shared meaning (8). Artistic practices—ranging from visual and performing arts to festivals, storytelling, and cultural events, among others—can engage multiple senses, bridge disciplinary boundaries, and invite audiences into reflection and dialogue, rather than didactic instructions (9, 10). Importantly, the arts can situate climate change within lived experience, place, identity, and values, making it tangible and relational rather than technical and distant.

Emerging scholarship suggests that arts-based approaches may be well-suited to address the affective and social dimensions of climate change, including coping with climate emotions, such as eco−/climate anxiety, distress and grief, towards radical hope and agency (11–13). By creating spaces for emotional expression, imagination, and communal meaning-making, the arts may support awareness and understanding, as well as psychological adaptation, resilience, and motivation for action (14). Furthermore, arts-based initiatives delivered in public and outdoor spaces have the potential to reach audiences beyond those already engaged with environmental or cultural institutions, supporting inclusion and social participation in climate-related reflection and dialogue (15–17).

International climate convenings have amplified the need to integrate the arts into climate action. UNESCO’s meeting on Culture, Heritage, and Climate Change (2021) marked a milestone in bridging culture, climate and health. Momentum continued at COP27 (2022), where the Climate Heritage Network launched the Sharm El-Sheikh Declaration on Culture-Based Climate Action, urging governments to enhance collaborations between culture and environment ministries. In COP28 (2023), the Group of Friends of Culture-Based Climate Action and the Emirates Declaration on Culture-Based Climate Action were established; an advocacy milestone for integrating arts and culture into global health and climate frameworks. Nevertheless, gaps remain between the proliferation of high-level declarations and the implementation of formal policy instruments. Although symbolic commitments are increasing, they have yet to be consistently reflected in multilateral climate mechanisms and policies (18).

While conceptual and theoretical discussions of climate–arts intersections are expanding, a need remains to synthesise current empirical evidence exploring how arts-based practices have been implemented, evaluated, and experienced in relation to climate communication, adaptation, and mitigation, with attention to both human and planetary health outcomes. To date, much of the literature remains fragmented across disciplines, methodologies, and art forms, making it difficult to assess what is known, where gaps remain, and how future initiatives might be designed to maximise long-term impact.

This scoping review aimed to identify, synthesise, and analyse empirical studies that have implemented arts-based interventions to address climate change communication, adaptation, and mitigation. Specifically, we sought to answer three research questions:

- How can the arts improve communication and raise awareness of climate change?
- How can the arts support adaptive behaviors and responses to climate change?
- How can the arts mitigate the effects of climate change on human and planetary health?

## 2 Methodology

We followed the scoping review methodology set out by Arksey and O’Malley (19) to identify the relevant studies, chart the data, analyse and report the review findings.

### 2.1 Identification and selection of studies

Identification of studies related to the arts: we searched for arts-based interventions and practices using terms related to the five main forms of art (20, 21), specifically: performing arts; visual arts, design and craft; community and cultural festivals; literature; online, digital, and electronic arts. We did not search explicitly for terms related to climate fiction, cinema, television or games, as the focus of the scoping review was on ‘live’, experiential, and co-created forms of art. However, this area has been explored in a previous and highly relevant integrative literature review (22).

Identification of studies related to climate: We used the following definitions and descriptions to identify studies related to climate communication, adaptation and mitigation:

Communication: We used the Yale Program on Climate Change Communication (23) two-fold description of communication: “*On the surface, climate change communication is about educating, informing, warning, persuading, mobilizing and solving this critical problem. At a deeper level, climate change communication is shaped by our different experiences, mental and cultural models, and underlying values and worldviews*.”

Adaptation: We used the Intergovernmental Panel on Climate Change (IPCC) (24) Glossary from the Sixth Assessment cycle, which refers to adaptation as “*the process of adjustment to actual or expected climate and its effects, in order to moderate harm or exploit beneficial opportunities*” in human systems and in natural systems.

Mitigation: We used the Intergovernmental Panel on Climate Change (IPCC) (24) Glossary from the Sixth Assessment cycle, which refers to mitigation as “*actions or activities that limit emissions of greenhouse gases from entering the atmosphere and/or reduce their levels in the atmosphere”*.

We searched for English-language publications without geographic or date restrictions, reflecting the pragmatic scope of a rapid scoping review. Eligible studies included qualitative, quantitative, and mixed-methods empirical research. We excluded non-empirical publications, such as opinion pieces, narrative articles, news articles, editorials, and commentaries, as well as unpublished or non-peer-reviewed master’s and doctoral dissertations, and conference abstracts or proceedings. Where relevant, these sources were drawn upon in the Introduction and Discussion to provide contextual background. This scoping review formed part of a larger study that also considered grey literature and global case studies, including evaluations of arts and culture programmes, guidebooks, news articles, and digital media. As many of these sources did not employ rigorous research methodologies and were therefore not eligible for inclusion in the present review, their findings were synthesised and presented separately (18).

### 2.2 Data collection

The scoping review comprised records from five major electronic databases (PsycINFO, PubMed, MEDLINE, CINAHL, and Cochrane Libraries); searches were completed on the 26th of August 2024.

Our search strategy included three steps:

- Step 1: Combined terms related to the arts, such as visual arts, performing arts, creative arts, expressive arts, music, dance, movement, drama, psychodrama, theater/theatre, poetry, play, storytelling, photography, digital arts, public arts, exhibitions and festivals.
- Step 2: Combined terms related to climate, such as climate communication, adaptation, mitigation, education, literacy, awareness, engagement, resilience, justice, action and activism.
- Step 3: Combined search 1 and 2.

For a detailed list of all terms searched, please see Table 1.

**Table 1:** Database search strategy.

‘Arts’ terms combined with OR	Combined terms	‘Climate’ terms combined with OR
Arts	Combined with AND	Climate
Visual arts		Climate crisis
Fine arts		Climate emergency
Performing arts		Climate action or activism
Expressive arts		Global warming
Creative arts or creativity		Climate communication
Music		Climate education
Song or singing		Climate engagement
Dance		Climate adaptation
Movement		Climate mitigation
Theatre or theater		Climate resilience
Drama		Climate justice
Psychodrama		Climate literacy
Performance		Climate information
Play		Climate awareness
Poem or poetry		Climate mobilisation or mobilization
Storytelling		Climate anxiety or distress or grief
Photography		Eco or ecology or ecosystem
Painting		Eco anxiety or distress or grief
Drawing		Environmental change
Sculpture or sculpting		Environmental protection
Craft		Environmental conservation
Festival		Outdoors or outdoor environment
Exhibition		Nature or natural environment
Digital arts		Planet or planetary health
Public arts		Biodiversity
		Net zero
		Sustainability

Two trained reviewers (ZM, MN) screened each record at both title-and-abstract and full-text levels using the eligibility criteria guide. Any uncertainties were resolved through discussion with a third member of the team until consensus was reached.

In total, 16,508 records were retrieved. Following title-and-abstract screening and removal of duplicates, only 66 records were deemed eligible for screening, suggesting that we used broad terms in our search strategy. For example, the term ‘play’ retrieved mainly games which were beyond the scope of this review, ‘movement’ retrieved mainly outdoor physical activities, and ‘environment’ retrieved thousands of irrelevant papers related to education, healthcare and workplace environments. From the remaining records, 16 were deemed eligible for inclusion in this scoping review. Some authors have published about the same work in more than one article; these articles were merged in the results for better clarity (see Tables 2, 3). The PRISMA diagram (Figure 1) details the study selection process.

**Table 2:** Study description.*

Authors/year	Title	Journal	Primary aim	Practice description**	Primary category***	Key findings
Baldwin and Chandler (33)	“At the water’s edge”: Community voices on climate change	Local Environment: The International Journal of Justice and Sustainability	To give voice to communities regarding the threats of climate change and rising sea levels on coastal communities.	Photovoice during a 10-day environmental art event called ‘Floating land’.	Communication	56% of respondents said that the project raised their climate change awareness.
						91% said that increased their understanding of others’ views.
						77% said that the event affected their interest in climate change.
Curtis (39), and Curtis (42, 43)	Creating inspiration: the role of the arts in creating empathy for ecological restoration	Ecological Society of Australia	To encourage emotional affinity with the natural environment through environmental arts events	Variety of environmental arts events, including ecological conferences, choirs, performances, exhibitions among others.	Communication	Environmental arts events: Affected pro-environmental beliefs, values and attitudes Raised awareness of consequences of actions Simplified and conveyed complex ecological and scientific information Formed pro-environmental social norms Built community involvement Reduced situational constraints and barriers to adopting pro-environmental behavior In one of the studies (29), based on 239 survey responses (2012), ~50% said that the arts encouraged them to reflect on alternative ways to communicate science. Yet only 24% would consider using the arts in their future work.
Curtis, et al. (29)						
Curtis, et al. (35)						
	Plague and the Moonflower: a regional community celebrates the environment	Music and Arts in Action				
	Using the arts to raise awareness and communicate environmental information in the extension context	Journal of Agricultural Education and Extension				
	Communicating ecology through art: what scientists think towards ecological sustainability: observations on the role of the arts					
Jacobson et al. (31)	Integrated science and art education for creative climate change	Journal of Ecology and Society	To develop new ways of viewing and interpreting environmental issues and develop more effective communication techniques.	An interdisciplinary field trip to a remote marine lab for graduate students from fine arts and natural resource science departments.	Communication	Increased self-awareness of students’ own environmental behavior and fewer misconceptions about climate change.
						Students saw climate change as more controversial than they had originally thought.
						Students provided novel images to communicate the impacts and conditions of mitigation and adaptation to climate change.
Marks et al. (34)	Re-imagining the environment: using an environmental art festival to encourage pro-environmental behavior and a sense of place	Local Environment: The International Journal of Justice and Sustainability	To encourage pro-environmental behavior, environmental awareness and stewardship, and a sense of place.	Environmental art festival called 'Floating Land'	Communication	41% intended to change their environmental behavior (some clarified that they had already adopted pro-environmental behaviors and had no intention to change that).
						No difference was found between participants with different demographic characteristics, or factors such as length of time spent at the festival, previous visits, and form of participation (as an artist, volunteer, organiser or observer).
						30% said that the most memorable aspect was the influence of the facilitators/artists.
						75% felt prouder for their community and 53% felt that they were part of the community.
Nyhan et al. (30)	Creative C-Change: analysing the impact of the Creative Climate	Cork Open Access Archive (CORA), University of Cork	To increase climate change awareness engagement and action in Ireland.	MaREI Centre for Energy, Climate & Marine and the Future Sustainability Research Group’s engagement with five Creative Ireland-funded Creative Climate Action projects	All categories	Significant impacts on environmental awareness and motivation to take climate change action.
	Action Initiative on climate change awareness, engagement and action in Ireland.					Majority of participants said that the artistic / creative events inspired them to take environmental action and planned to change their behavior and lifestyle as a result. Events fostered community spirit and gave people a sense of purpose.
Riechers et al. (36)	Stories of favourite places in public spaces: emotional responses to landscape change	Sustainability	To explore emotional responses towards landscape change and elicit an intervention that empowers local communities through social land art.	Land-use and nature conservation through interactions with local actors.	Adaptation	‘Resilient’ emotional responses towards landscape change showed higher will to integrate modifications needed, while ‘non-resilient’ responses showed lower will, frustration and despair.
						Authors argued that identifying emotions towards change are crucial to foster collective knowledge generation, use discomfort as a motivational source for transformation, and to prevent a spiral of disengagement and apathy through participatory processes.
Sommer and Klöckner (27)	Does activist art have the capacity to raise awareness in audiences? A study on climate change art at the ArtCOP21 Event in Paris	Journal of Psychology of Aesthetics, Creativity,	To explore the effect of environmental artworks in terms of emotional activation, cognitive activation, and characteristics of the artwork	Analysis of 37 visual artworks presented in ArtCOP21 (21st United Nations climate summit in Paris).	All categories	Environmental artwork should move away from a dystopian way of depicting the problems of climate change, toward offering solutions, and emphasizing the beauty and interconnectedness of nature.
Klöckner and Sommer (26)	Visual art inspired by climate change: analysis of audience reactions to 37 artworks	Plos One	To explore the psychological mechanisms by which climate change-related art might relate to people’s reflections on climate change and support for climate policies and mitigation.			Being confronted with climate change-related artwork links to increased (at least short-term) support for climate policy.
						Reflections were higher in artworks that challenged social norms and felt more personal.
						Feelings were associated with artwork characteristics (e.g., colors, size, design). For example, positive emotions were found if the artwork included something personal, was not too big, presented with an explanation of how to "read", but did not include destruction, especially of known landmarks. Negative emotions were triggered by polarizing and monumental artworks. However, both negative and positive emotions elicited support for climate policy and action.
Woodland et al. (37, 40)	Embodying resistance and survival: how drama and youth theatre can respond to eco-anxiety and support recovery in disaster affected communities	Creativity and Wellbeing Hallmark Research Initiative (CAWRI), University of Melbourne.	To examine the role of youth theatre and performance in responding to the multifaceted effects of climate change and eco-anxiety, and to experiences of trauma and loss within disaster-affected communities.	Youth theater and performance for post-disaster recovery and eco-anxiety experienced by young people in Australia.	Adaptation	Young people tended to perform about ‘resistance and survival’ in response to disasters and the climate crisis.
						Findings led to the development of a framework to understand how young people cope with climate crisis across four intersecting domains: disaster preparedness, first response, disaster recovery, and climate activism. The ways that performance works across these domains traverses artistic practice and everyday actions that contribute to young people’s sense of hope, agency, and community.
						Theater and performance were found effective in disaster preparedness, disaster recovery, and climate activism.
		Research in Drama Education: The Journal of Applied Theatre and Performance				
Woodley et al. (28)	Climate Stories: enabling and sustaining arts interventions in climate science communication	Geoscience Communication	To mobilise the arts to provide more engaging narratives of climate change.	A 3-day residential retreat called ‘Climate Stories’. It built on the UK’s WAMfest (Weather, Arts and Music Festival) to facilitate climate communication through printmaking, songwriting, theatre, performance, and festivals open to the public.	Communication	Climate scientists’ growing personal and professional communication confidence, and reconnection with the creative dimensions of their professions. They also experienced and reported a sense of liberation from more rigid communicative frameworks.

*Publications from the same authors that describe the same practice have been combined for clarity.

**Detailed practice description is presented in Table 3.

***Category: Communication, Adaptation, Mitigation.

**Table 3:** Practice description.

Author/year	Country	Type of arts	Active vs receptive arts engagement	Participants	Practice evaluation
Baldwin and Chandler (33)	Australia	Visual arts	Active	16 participants, including 4 artists, 8 community members/residents, and 4 event visitors.	Photovoice in 3 stages:
					1. Participants took photos to represent what they value about being at the water’s edge; the perceived threats; and what they value about the Floating Land event.
					2. Participants selected 9 pictures to shape the focus group.
					3. Participants completed a survey to understand whether the event stimulated curiosity, interest and awareness around climate change, and motivation towards action.
Curtis (39) and Curtis (42, 43)	Australia	Mixed	Active and receptive	Number of interviewees varied in each study, from small (*n* = 24) to large sample sizes (*n* = 239).	Interviews with various groups, including artists, performers, musicians, audience, extensionists and natural resource management scientists.
Curtis et al. (29)					
Curtis et al. (35)					
Jacobson, et al. (31)	Florida, USA	Visual arts	Active	Two artists and two biological scientists facilitated the fieldtrip.	Pre-and-post survey, focus groups, artwork critiques by art and communication experts.
				Participants were 18 students from fine art and natural resource management classes.	
Marks et al. (34)	Australia	Mixed	Active and receptive	120 audience respondents	Pre-and-post festival questionnaires from the audience, workshop participants, and community members/residents.
				30 workshop respondents	
				60 pre-festival resident respondents	
				36 post-festival resident respondents	
Nyhan et al. (30)	Ireland	Mixed	Active	300 public participants aged between 35 and 44 years’ old.	Surveys, interviews, participant observations, artist reflections and workshops.
					Survey included the EPA’s Climate Change in the Irish Mind survey and Eurobarometer surveys.
				61% females, 38% males, 1% preferred not to say.	
Riechers et al. (36)	Germany	Mixed	Active	Eight participants, including the artist and the researcher	Audio recordings, participant observations, workshops, and 17 additional interviews with local experts.
				5 females/3 males	
Sommer and Klöckner (27) and Klöckner and Sommer (26)	Paris, France	Visual arts	Receptive	883 responses from the public	Questionnaires based on multiple environmental psychological theories.
					Measures of emotional reaction, perceived quality, identification with the artist, artwork reflections, climate policy support, and other artwork characteristics.
				56% females (*n* = 490)	
				44% males (*n* = 382)	
				Mean age: 36.62 (SD 16.43)	
Woodland, et al. (37, 40)	Australia	Performing arts	Active	12 youth theater and performance practitioners (e.g., from grassroots youth community groups, arts companies, circus, immersive performances, multi-arts festivals).	Individual interviews.
Woodley et al. (28)	United Kingdom	Mixed	Active	19 climate scientists from the Met Office and the University of Exeter.	Reflective diaries, interviews, workshops.
				Participants ranged from postgraduate students to senior climate scientists; most were at an early career stage.	

**Figure 1:**
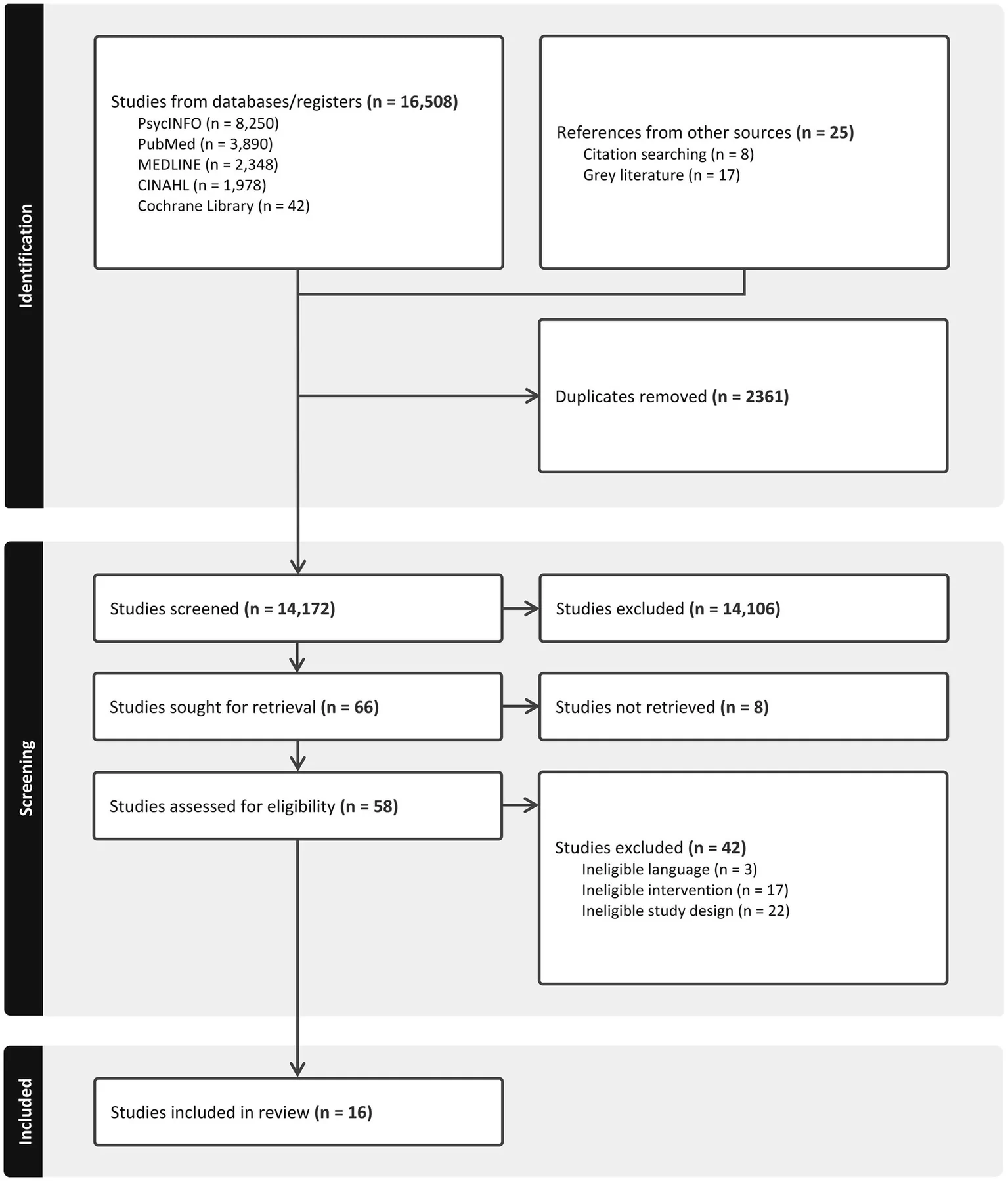
PRISMA flowchart.

### 2.3 Data charting and analysis

Five authors (ZM, MN, EB, TB, SM) collectively determined the information to extract from all records. We charted descriptive details, such as art form, country, and research design. The chart also included details such as the length and intensity of arts engagement, study objectives and whether these objectives were achieved, and how this was evaluated (25).

Following an iterative process, the same five authors developed, piloted, and updated the data charting form to determine if the approach to data extraction was consistent with the research question and scoping review purpose. The data from each paper was extracted by the lead author (ZM), with the team meeting regularly to discuss the process. Data synthesis and preparation of the final manuscript were led by the first author (ZM), followed by iterative rounds of revision and critical review by all co-authors.

## 3 Results

We first describe demographic characteristics of included studies, followed by addressing each research question in detail. Some authors published about the same work in more than one article; these articles have been merged in the results and tables for better clarity (see Tables 2, 3).

### 3.1 Demographics findings

Types of arts: Sixteen studies were included covering a range of programs such as environmental art festivals, theater and open-air performances, exhibitions, and musical events. Most studies adopted both visual and performing arts (*n* = 9), some visual arts only (*n* = 4), and one study utilised performing arts only.

*Active vs. receptive arts engagement*: There was a combination of active and receptive engagement with the arts. Active engagement included directly making, performing, or creating art. Examples included painting a picture, writing a poem, making a movie, or acting in a play. Receptive engagement included experiencing, attending, listening, or viewing art. Examples included viewing a painting in a gallery, reading a poem, watching a movie, or attending a performance. Most studies actively engaged participants with the arts (*n* = 6), some used a combination of active and receptive arts engagement (*n* = 2), and one study utilised perceptive engagement only.

Countries: The studies were conducted in six countries, specifically: Australia (*n* = 9), France (*n* = 2), USA (*n* = 1), Ireland (*n* = 1), Germany (*n* = 1), and the UK (*n* = 1). Representation from low- and middle-income countries was missing as well as international collaborations.

Study aims: Most studies focused on climate change communication and public health awareness (*n* = 9); only a small number of studies focused on adaptation (e.g., coping with climate emotions) (*n* = 2) or mitigation (e.g., motivating cultural shifts towards low-carbon lifestyles and practices) (*n* = 2).

Sample size: The overall known sample was 1,645 participants. Some studies included participants from multiple events (e.g., studies led by Curtis), whereas others focused on single events or activities. The largest study included data collected from 883 participants across a series of events during a UN climate summit (26, 27). Demographic characteristics of the participants and dropout rates were rarely reported.

### 3.2 Research question 1: how can the arts improve communication and raise awareness of climate change?

Most included studies focused on the role of the arts in communicating about and raising awareness of the effects of climate change on health. This is a crucial area considering that climate scientists face major communication challenges due to the heavily politicised landscape that is characterised by denial, scepticism, distrust, and increased prevalence of misinformation (28). Conventional communication approaches, such as reports, publications and presentations, may increase the distance between scientists and the public, particularly for people living in areas where climate change is not perceived to pose a direct threat yet.

#### 3.2.1 Communicating and raising awareness

Existing evidence demonstrates that engagement with the arts can improve communication and raise awareness of climate change. This may be because the arts can simplify, synthesise, and convey complex ecological and scientific information, as well as abstract concepts, making them more accessible and comprehensible (29).

In Nyhan et al. (30) Creative Climate Action projects funded by Creative Ireland, authors found a significant change in awareness of climate change matters, with 82% of the participants saying that the artists and the creative process highlight the impact of climate change better than facts alone. In addition, 83% said that they understand what actions they should take to address climate change.

In Jacobson et al. (31), an interdisciplinary field trip to a remote marine lab, attended by graduate students from fine arts and natural resource science departments in the University of Florida, USA, led to statistically significant changes in students’ knowledge and realisation of the complexity of climate change. For example, students found climate change more controversial among climate scientists than they had originally thought. Interestingly, authors also found that the field trip led to fewer misconceptions, which is the only data found in this review suggesting that the arts can address misinformation.

In Woodley et al. (28) Climate Stories in the UK, authors found that when climate scientists worked alongside artists, they experienced increased personal and professional communication confidence, and reconnected with a creative dimension of themselves that was professionally suppressed through long-term adherence to scientific protocols and conventions (32).

In Baldwin and Chandler (33) Floating Land art festival in Australia, although only 56% of the participants said that their awareness of climate change increased, 77% said that the event affected their interest in climate change, and 91% reported that it increased their understanding of others’ viewpoints.

#### 3.2.2 Generating novel perspectives through interdisciplinary lens

Climate change requires knowledge and understanding from several disciplines, which is one reason why climate change is often difficult to comprehend. The collaboration between experts from different disciplines, such as artists, scientists, psychologists, healthcare professional, commissioners and policymakers, created the conditions needed to understand climate change more holistically. Engagement with the arts generated new and novel perspectives about climate change that had not been considered before.

In Nyhan et al. (30) Creative Climate Action projects in Ireland, 66% of the participants said that the projects helped them to interact with environmental and climate change issues in new ways, while 76% of the participants said that the projects helped them to interact with the arts, heritage, and cultural organisations in new ways. These novel perspectives were largely attributed to the participants’ engagement with artists, which came with an acknowledgement and appreciation of the skills that artists bring. As one of the artists in this project said:

“*As an artist, you are a witness and you’re an observer, documenter, a recorder, a magician, a healer, social worker, a reflector, quasi-academic, quasi-social work, quasi-politician, and producer, you know?*”

In Jacobson et al. (31), students said that the field trip to the remote marine lab in Florida, USA, provided them with new, novel, and more diverse images to translate climate change knowledge. The authors argued that the arts can make natural resource scientists more effective communicators and normalise different ways of viewing. As one of the natural science students said: “*[The art students] look at the world in a whole different way.*”

In Marks et al. (34) Floating Land art festival in Australia, 30% of the participants echoed that the influence of the facilitators/artists was their most memorable aspect of the festival.

#### 3.2.3 Aesthetic beauty and pleasure

Making climate change matters more ‘aesthetically pleasing’ increased accessibility and the public’s engagement both with the arts and with climate change. In this context, ‘aesthetically pleasing’ refers to observable characteristics shown to elicit emotional and cognitive responses, such as through visual organisation and accessibility of meaning and interpretation. Regardless of the arts medium that was utilised, existing evidence demonstrates that aesthetics and the sense of wonder increased people’s engagement and participation in the arts events (35).

Most significant evidence on aesthetics comes from two articles by Sommer and Klöckner (27) and Klöckner and Sommer (26), who analysed the impact of Climate Change Art at the ArtCOP21 Event in Paris in 2015. In their first article, Sommer and Klöckner (27) explored how specific features of the artwork relate to how strongly and which emotions are activated by the audience. They divided their artwork features into four categories, with each category resulting in different emotional responses.

- The ‘comforting utopia’ referred to playful and colorful ways to visualise a ‘utopia’ or a better future. Artwork with such characteristics were perceived as low artistic quality or oversimplistic. Although these artworks elicited emotions of hope and happiness, rather than guilt and sadness, they led to only minimal degrees of cognitive activation, confrontation, reflection, and awareness of one’s own behaviors. No contribution was found towards a sense of belonging.
- The ‘challenging dystopia’ referred to dark colors that represent dystopian scenarios of death and destruction. Such features produced negative emotions, reduced sense of belonging, and did not trigger reflection or inspire people to act.
- The ‘mediocre mythology’ referred to a colorful mix of materials with themes of mythology and depictions of Earth. Such features evoked neutral responses (i.e., not shocking but not exciting either) and did not contribute to emotional activation.
- The ‘awesome solution’ referred to colorful depictions of sublime nature that showed solutions to environmental problems. This kind of artwork was often observed in outdoor environments and public spaces. These features were the most effective in eliciting high cognitive activation, reflection, and awareness of the impact of individuals’ behavior on climate change. Importantly, artwork in this category did not just present a problem but also offered creative solutions.

Based on the analysis of these features, the authors concluded that environmental artwork should shift away from dystopian ways of presenting climate change towards gently offering solutions, while also emphasising the beauty and interconnectedness of nature.

In their second article, Klöckner and Sommer (26) explored whether positive or negative feelings were associated with specific artwork characteristics, such as color, size, design, depiction of known objects, and presence of destruction (among other characteristics), as well as which of these characteristics were the most impactful. They concluded that more positive emotions were found in artwork that included something personal, was not too big, and was presented with an explanation of how to ‘read’ it, and without ‘destructions’ especially of known landmarks. Negative emotions, on the other hand, were more likely triggered by polarizing and monumental artworks. These findings are important in understanding how aesthetics can affect people’s willingness to take climate action.

Findings suggesting that the beauty of environmental artworks is more impactful when exhibited in outdoor public or natural spaces was also echoed by Marks et al. (34). They found that exhibiting artwork in public outdoor or natural environments drew audiences’ attention to the beauty of their everyday surroundings, thereby enhancing their appreciation of Nature and strengthening their sense of place. This resulted in a higher motivation to adopt pro-environmental behavior and take climate action; findings that were echoed in a similar systematic review of arts-in-nature interventions (13, 17).

#### 3.2.4 Encouraging reflection

Every study included in this review found that the arts made people more reflective towards their own environmental attitudes and behaviors.

Jacobson et al. (31) found that students’ awareness of their own environmental behavior was significantly increased following their fieldtrip. Nyhan et al. (30) also found that:

- 85% reflected upon the relationship between humanity and nature.
- 77% understood better the connection between their actions and the health of the planet.
- 70% realised how addressing climate change would help tackle health inequalities.
- 69% understood the consequences for their community of taking no action on climate change.

Klöckner and Sommer (26) reported that some artwork features increased the level of reflection. These features were about challenging social norms and including something personal for the audience to identify with. As such, it appears that what makes environmental arts impactful is the emotional and personal connection they can create to a topic that, at times, feels distant and abstract.

### 3.3 Research question 2: how can the arts support adaptive behaviors and responses to climate change?

The mechanisms by which adaptation was achieved through the arts varied across the studies, but the most mentioned mechanisms are presented below.

#### 3.3.1 Generating both cognitive and emotional activation

Sommer and Klöckner (27) argued that emotional activation alone is not enough when people are not aware of what actions they need to take. Vice versa, cognitive or intellectual activation alone is not enough when people are not emotionally invested in climate action. The combination of both is necessary to adapt to and mitigate the health impacts of climate change. The authors acknowledged that cognitions and emotions do not exist separate from each other, and the order in which they are triggered is often hard to define. However, they explored some of the mechanisms by which cognitive and emotional activation can be achieved in the process of art viewing or art making:

- The cognitive processing, referring to the question ‘What does this artwork mean/tell me?’
- The motivational processing, referring to the question ‘Does the artwork motivate me to a certain action?’
- The physiological reactions, referring to responses such as sweat, heart rate, or chest tightness during art viewing or art making.
- The behavioral reactions, referring to actual behavior changes achieved as a result of art viewing or art making, such as cycling to work or reducing meat-based meals.

#### 3.3.2 Processing better the cognitive and emotional activation

The combination of new knowledge and arising emotions related to climate change can be overwhelming. Some authors therefore explored how the arts can contribute towards better processing of cognitive activation (e.g., dealing with advanced climate literacy or awareness) alongside processing of emotional activation (e.g., facing more complex climate emotions).

Riechers et al. (36) explored the emotional responses of local communities towards landscape change in an intensively farmed landscape in Germany. Through social land art, participants in this study expressed a spectrum of emotional responses, ranging from ‘resilient’ incorporation of changes to ‘non-resilient’ reactions, such as frustration, anger and despair. The use of arts for emotional expression and processing gave space to dialogue and knowledge generation about what actions can be taken collectively. Authors found that even the ‘non-resilient’ responses, such as discomfort, can be a motivating source for action and community transformation. They concluded that creative participatory processes such as social land art can prevent the spiral of disengagement, apathy, and despair, and lead to meaningful climate adaptation.

Woodland et al. (37) implemented youth theater and performance to rehearse disaster preparedness, recovery, and adaptation for young people who experience eco-anxiety in Australia. The authors found the rehearsal and performances effective not only in alleviating eco-anxiety but also making young people feel better prepared for future disasters and more willing to contribute towards climate activism.

#### 3.3.3 Raising inspiration, hope, and empowerment to adapt

Several authors argued that the arts raised people’s inspiration, hope, and empowerment to adapt to climate change; however, this was mostly demonstrated through qualitative data. The primary study that reported quantitative data was by Nyhan et al. (30) Creative Climate Action projects in Ireland. The authors found that:

- 95% said that artistic/creative events inspired them to take positive environmental action.
- 74% planned to make changes to their behavior or lifestyle.
- 70% felt empowered to take climate action.
- 85% said that addressing climate change would provide people with a sense of purpose.

Nevertheless, whether inspiration, hope, and empowerment to adapt were enough to lead to actual adaptations remains unknown and was not explicitly explored in the included studies.

#### 3.3.4 Sense of community and belonging through solidarity in shared values

Arguably the most mentioned aspect of climate adaptation was the ability of the arts to unite and bring people closer through solidarity and commitment to in shared values. This process was described by some authors as ‘resonance’, whereby the artwork’s personal and cultural significance helps people to “*feel part (or imagine being part) of a shared cultural space*” (38).

Marks et al. (34) also found that 75% of participants felt prouder of Boreen Point (Australian community), 53% felt more part of the community, and 47% wanted to do more for their community.

In Nyhan’s et al. (30) study, survey responses showed that:

- 88% agreed that addressing climate change fosters greater community spirit.
- 85% agreed that addressing climate change provides people with a sense of purpose.
- 79% agreed that they made new connections or developed existing relationships within their local community.
- 67% felt a greater sense of appreciation and pride in their community.

In one of the qualitative studies (39), a member of the audience echoed these findings by saying that: “*I’ve lived in Armidale for 25 years [and] I’ve never been more proud to come from this community.*”

### 3.4 Research question 3: how can the arts mitigate the effects of climate change on human and planetary health?

Literature exploring the role of the arts in mitigating the climate effects on human and planetary health is limited. We identified only three primary contributions as presented below.

#### 3.4.1 Policy engagement and influence

Arts engagement appeared to make the public more willing to engage with policymakers. For example, in Nyhan’s et al. (30) study, 55% of the participants said that the Creative Climate Action projects instilled in them a willingness to lobby politicians on climate change.

Klöckner and Sommer (26) found that being confronted with climate change-related artwork relates (at least in the short-run) to increased climate policy support and advocacy. Interestingly, they found that the support for climate policies was stronger among people with previously ‘weak’ pro-environmental attitudes than for people with already ‘strong’ pro-environmental attitudes. Equally surprising was that the support for climate policy action was elicited regardless of whether the artwork resulted in ‘positive’ or ‘negative’ emotions towards climate change.

#### 3.4.2 Creative action and activism

Several studies argued that their projects resulted in increased interest in creative action and activism. However, we did not find sufficient data to assess whether this was the case. In Woodland et al. (40), one of the four primary themes was young people’s willingness to engage with youth climate activism following their participation in the project. In Nyhan et al. (30), 67% of the participants said that their participation in the Creative Climate Action projects helped them to interact with environmental and climate change organisations, therefore the authors concluded that the projects enhanced bottom-up activism.

#### 3.4.3 Pro-environmental attitudes and behaviors

There is inconclusive data as to whether the programmes or interventions supported the development of pro-environmental attitudes and behaviors. In Nyhan et al. (30), over half of the participants (74%) said that they were planning to make significant lifestyle changes to mitigate the effects of climate change on health. These changes included planting for biodiversity and creating better green spaces, reducing household waste and recycling more, repairing damaged possessions instead of replacing them, reducing consumption of disposable items, purchasing less non-essential items, and buying second-hand goods where possible. However, in Marks et al. (34), less than half of the participants intended to change environmental behavior. This finding was consistent across participants regardless of demographic characteristics or the degree and length of involvement in the festival. Interestingly, the intention to change behavior was higher for people who came to the festival without previous environmental intentions.

## 4 Discussion

This scoping review identified sixteen empirical studies examining the role of the arts in climate change communication, adaptation, and mitigation. They covered a range of programs such as art festivals, climate summits, ecological conferences, theater and open-air performances, exhibitions, musical events, field trips, nature conservation and sustainability activities. Collectively, the findings highlight the considerable promise of arts-based approaches for engaging diverse publics with climate change, while also revealing important gaps in scope, evaluation, and long-term impact. Most studies focused on climate change communication and awareness-raising, with comparatively fewer addressing adaptation (e.g., coping with climate emotions) or mitigation (e.g., motivating cultural shifts towards low-carbon lifestyles and practices). This imbalance suggests that while the arts are increasingly recognised as powerful communicative tools, their potential to support sustained behavioral change, psychological adaptation, and structural mitigation remain underexplored.

### 4.1 The arts as a catalyst for climate communication

Across studies, the arts consistently demonstrated an ability to make climate change more accessible, tangible, and personally relevant. By translating scientific concepts into visual, embodied, and narrative forms, arts-based practices helped participants better understand the complexity of climate change and reflect on the implications for their own lives, communities, human and wider planetary health. This capacity to ‘emotionalise’ without resorting to fear-based or catastrophic messaging that overwhelms audiences can lead to cognitive and emotional activation (i.e., knowing what kind of action is needed and feeling emotionally invested in taking action) (27); this is important considering that the combination of the two is necessary for climate action.

Notably, studies analysing aesthetic qualities of climate-related artworks (26, 27) found that awe-inspiring depictions of nature and solution-oriented narratives were more positively received than dystopian, catastrophic, or overtly didactic narratives. Artwork that invited personal interpretation, emphasised beauty and interconnectedness, and gently challenged social norms, were more likely to elicit reflection, awareness of personal behavior, and openness to action. In contrast, artwork perceived as propagandistic or polarising often triggered disengagement and defensive responses. These findings highlight the importance of artistic autonomy and nuance, and caution against instrumentalising the arts solely as vehicles for predetermined advocacy messages.

### 4.2 Climate adaptation through cognitive, emotional, and social engagement

Although fewer in number, studies focusing on adaptation revealed important mechanisms through which the arts can generate both cognitive and emotional activation, empowerment, radical hope, and solidarity in shared values. Participatory arts practices created spaces for emotional expression, processing, dialogue, and meaning making around climate distress, loss and uncertainty. By legitimising a range of emotional responses, including discomfort, grief, and anger, the arts helped both adults and young people to process climate emotions, and transform distress and withdrawal into motivation and empowerment for collective climate action (37).

Several studies highlighted the role of the arts in fostering a sense of community, belonging, and purpose. While much of this evidence was qualitative, survey findings demonstrated higher levels of self-reported inspiration and intention to act (30); pointing to the potential of artistic engagement to mobilise communities by transforming how individuals understand and engage with climate issues and by fostering the attitudinal and behavioral shifts required for transformative climate action. The arts also strengthened social bonds and collective identity, reinforcing a sense of belonging and solidarity in shared values (34, 39). This social dimension is critical, as adaptation to climate change is not solely an individual endeavour but a collective one, requiring community cohesion, trust, solidarity, and a shared vision.

### 4.3 Climate mitigation through behavior change, policy engagement and creative activism

Evidence regarding the role of the arts in climate mitigation was limited and mixed. Some studies reported increased intentions to adopt pro-environmental behavior or support climate policies (30, 31, 40), particularly among participants who did not previously hold strong environmental attitudes (26). This suggests that arts-based approaches may be especially effective in reaching ‘new’ audiences and broadening public engagement. Nevertheless, there was little evidence documenting whether these intentions translated into sustained behavior change or measurable changes in environmental impact.

Engagement with policy and advocacy emerged as a notable, though underdeveloped, outcome. Exposure to and involvement with the arts was associated with increased willingness to engage with policymakers and support climate action, at least in the short term (26). These findings point to the arts’ potential role as a catalyst for civic participation, social inclusion, and democratic engagement in climate governance, especially for communities and people who have been traditionally excluded from decision-making processes. Nevertheless, the absence of longitudinal data limits conclusions concerning durability and real-world impact.

### 4.4 Implications for research, practice, and policy

There are several implications for advancing the role of the arts in climate communication, adaptation and mitigation. First, future initiatives should move beyond treating art as a tool for translating scientific knowledge (41), and instead embrace its capacity to generate dialogue, challenge assumptions, and open imaginative space for alternative futures (28). As such, artwork should avoid carrying explicit reference to specific advocacy positions (28) or instill didacticism and propagandization (27). Successful collaborations require respect for artistic processes, tolerance for ambiguity, and a balance between structure and spontaneity.

Second, there is a need to extend arts-based initiatives beyond institutional settings into public and outdoor spaces. Doing so can democratise access, reduce perceptions of elitism, and root climate issues more visibly within everyday lived environments (27). Embedding artwork in natural settings may also cultivate a deeper appreciation for ecological beauty and strengthen people’s sense of place (13, 17, 34). Crucially, when climate action is tied to a specific location, it may be experienced as more concrete and tangible (33).

Third, universities, cultural institutions, government and funding bodies should develop structures that enable and reward cross-disciplinary collaboration, particularly between the arts and sciences. Institutional frameworks should recognise advocacy and public engagement as legitimate scholarly contributions, valuing art–science partnerships alongside conventional outputs (28). Higher education can establish structures that foster teacher and student development through cross-curricular innovation. For example, natural science students can learn creative methods of communicating climate issues from arts students, while arts students can deepen their understanding of ecological issues and create environmental artwork through collaboration with natural science students (31).

Finally, future research should address methodological limitations that were present throughout this review. Most studies relied on small, self-selecting samples and short-term evaluations, with limited reporting of intervention characteristics. Longitudinal mixed-methods research is needed to examine sustained behavioral change, the connection between human and planetary health outcomes, and the extent to which creative climate initiatives contribute to policy action and measurable impact.

### 4.5 Limitations

Several limitations should be considered when interpreting the findings of this review. The evidence base was relatively small and geographically concentrated, with most studies conducted in high-income, English-speaking countries. The restriction to English-language publications may have further reinforced this geographical and cultural bias, limiting the transferability of the findings to other cultural, linguistic, and socioeconomic contexts. Although this scoping review formed part of a broader study that also examined grey literature and global case studies, including evaluations of arts and culture programmes, guidebooks, news articles, and digital media, many of these sources did not employ sufficiently rigorous research methodologies to meet the inclusion criteria for the present review. Their findings were therefore synthesised and reported separately (18).

Furthermore, the conclusions that can be drawn are constrained by the methodological limitations of the included evidence. Studies relied predominantly on qualitative, descriptive, and proxy-reported data, while considerable variation in study designs, populations, interventions, and outcome measures limited meaningful comparison and synthesis across studies. In addition, participants were often self-selected and may have had a pre-existing interest in the arts, culture, or climate-related issues, increasing the potential for selection bias and potentially limiting the generalisability of the findings.

These limitations highlight the need for a more robust, geographically and culturally diverse evidence base. Future research should employ rigorous and transparent methodologies, incorporate systematic evaluation and appropriate outcome measures, and examine the role of arts and culture across a wider range of cultural, socioeconomic, and geographical contexts.

## 5 Conclusion

This scoping review synthesised empirical evidence on the role of the arts in climate communication, adaptation, and mitigation. Across sixteen studies, the arts consistently emerged as powerful mechanisms for translating complex climate science into accessible, emotionally resonant, and socially meaningful experiences. Arts-based approaches were especially effective in raising awareness, encouraging reflection, fostering dialogue, and engaging audiences beyond those already invested in the arts and/or climate issues. In doing so, the arts helped bridge the persistent gap between knowledge and lived experience that continues to challenge conventional climate communication, adaptation and mitigation.

However, the review reveals a notable imbalance in the current evidence base. While arts-based initiatives for climate communication are increasingly visible, substantially fewer studies have examined their role in supporting adaptation and mitigation, including sustained behavior change and policy engagement. This represents a missed opportunity, particularly given the demonstrated capacity of the arts to support emotional processing, radical hope, collective identity, and civic participation; factors that are central to effective public health responses to climate change.

The findings suggest that the most impactful arts-based climate initiatives are those that combine cognitive and emotional engagement, avoid didactic or propagandistic framing, and offer audiences pathways toward imagining solutions rather than solely confronting loss or catastrophe. When delivered in public, outdoor, and community-based settings, the arts can also enhance inclusivity, strengthen sense of place, and support social cohesion; all of which are key determinants of health in a changing climate.

Realising the full potential of the arts in advancing climate communication, adaptation and mitigation requires stronger integration across research, policy, and practice. This includes sustained investment in interdisciplinary collaborations, institutional support for art–science partnerships, and evaluation frameworks capable of capturing long-term and combined human and planetary health outcomes. Future research should prioritise longitudinal designs and more representative samples, alongside a stronger focus on structural barriers that shape and constrain individual and collective climate action.

In the context of escalating climate-related health risks, the arts are not peripheral or supplementary, but essential health, cultural, and social resources within comprehensive climate and public health strategies. By connecting knowledge with emotion, lived experience, and shared values, the arts can strengthen individual and collective adaptive capacity and support more equitable responses to the interconnected challenges of human and planetary health.
